# Seasonal dynamics of tick burden and associated *Borrelia burgdorferi* s.l. and *Borrelia miyamotoi* infections in rodents in a Dutch forest ecosystem

**DOI:** 10.1007/s10493-022-00720-z

**Published:** 2022-07-16

**Authors:** Gilian van Duijvendijk, Inge Krijger, Marloes van Schaijk, Manoj Fonville, Gerrit Gort, Hein Sprong, Willem Takken

**Affiliations:** 1 Laboratory of Entomology, Wageningen University, PO box 16, 6700 AA Wageningen, The Netherlands; 2 Wageningen Environmental Research, Wageningen University and Research, PO box 47, 6700 AA Wageningen, The Netherlands; 3 Stichting Kennis- en Adviescentrum Dierplagen (KAD), Nudepark 145, 6702 DZ Wageningen, The Netherlands; 4 Koppert Biological Systems, Industrieweg 14, 2651 BE Berkel en Rodenrijs, The Netherlands; 5 Laboratory for Zoonosis and Environmental Microbiology, National Institute for Public Health and the Environment (RIVM), P.O. Box 1, 3720 BA Bilthoven, The Netherlands; 6 Mathematical and Statistical Methods, Wageningen University, PO box 16, 6700 AA Wageningen, The Netherlands

**Keywords:** *Ixodes ricinus*, *Apodemus sylvaticus*, *Myodes glareolus*, *Borrelia burgdorferi*, *Borrelia afzelii*, *Borrelia miyamotoi*, Seasonal dynamics

## Abstract

*Ixodes ricinus* ticks transmit *Borrelia burgdorferi* sensu lato (s.l.) as well as *Borrelia miyamotoi*. Larvae become infected when feeding on infected rodents, with horizontal transmission of *B. burgdorferi* and horizontal and vertical transmission of *B. miyamotoi*. We studied seasonal dynamics of infection rates of *I. ricinus* and their rodent hosts, and hence transmission risk of these two distinctly different *Borrelia* species. Rodents were live-trapped and inspected for ticks from May to November in 2013 and 2014 in a forest in The Netherlands. Trapped rodents were temporarily housed in the laboratory and detached ticks were collected. *Borrelia* infections were determined from the trapped rodents and collected ticks. *Borrelia burgdorferi* s.l. and *B. miyamotoi* were found in ticks as well as in rodents. Rodent density was higher in 2014, whereas tick burden as well as the *Borrelia* infection rates in rodents were higher in 2013. The density of *B. miyamotoi*-infected nymphs did not differ between the years. Tick burdens were higher on *Apodemus sylvaticus* than on *Myodes glareolus*, and higher on males than on females. *Borrelia*-infection rate of rodents varied strongly seasonally, peaking in summer. As the larval tick burden also peaked in summer, the generation of infected nymphs was highest in summer. We conclude that the heterogeneity of environmental and host-specific factors affects the seasonal transmission of *Borrelia* spp., and that these effects act more strongly on horizontally transmitted *B. burgdorferi* spp. than on the vertically transmitted *B. miyamotoi*.

**Supplementary Information:**

The online version contains supplementary material available at 10.1007/s10493-022-00720-z.

## Introduction

Lyme borreliosis is caused by an infection with *Borrelia burgdorferi* sensu lato (s.l.), which is, in Europe, transmitted by the sheep tick *Ixodes ricinus*. The same tick species also transmits *Borrelia miyamotoi*, which can cause Hard Tick-borne Relapsing Fever (HTRF). After hatching, *I. ricinus* larvae search for a vertebrate host. Rodents are commonly used as blood hosts by larvae and *B. burgdorferi* s.l. spirochaetes can be transmitted from a persistently infected rodent to feeding larvae (Hofmeester et al. 2016). Infected engorged larvae moult into infected nymphs, which can transmit the spirochaetes to rodents, thus closing the *B. burgdorferi* infection cycle among rodents. In contrast to *B. burgdorferi* s.l., *B. miyamotoi* is not only transmitted from transiently infectious rodents to larvae (Burri et al. 2014), but also transovarially in the ticks (Hauck et al. 2020; Scoles et al. 2001). The lifecycle of *B. miyamotoi* is, therefore, less dependent on rodents (Barbour et al. 2009). *Ixodes ricinus* can, at a low level, be co-infected with both pathogens (Kiewra et al. 2014; Kjelland et al. 2015). Ticks are highly aggregated among rodents and a minority of rodents feeds the majority of the ticks (Perkins et al. 2003), following the Pareto principle or the 20/80 rule (Woolhouse et al. 1997). A high burden of infected ticks increases the likelihood of rodents becoming infected with *B. burgdorferi* s.l., and a high larval tick burden on infected rodents increases the infection prevalence in questing nymphs. It is expected that the contribution of rodents to the density of infected nymphs is, therefore, also heterogeneous (Harrison and Bennett 2012; Krawczyk et al. 2020). The contribution of an individual rodent to the density of *B. burgdorferi*-infected nymphs is affected by (1) the number of larvae that feed on the rodent (larval tick burden), (2) the chance that the rodent is infected (rodent infection rate), (3) the chance that the spirochaetes are transmitted to feeding larvae (rodent infectivity), and (4) the chance that the engorged larvae moult into nymphs (moulting success). As the transmission route of *B. miyamotoi* goes via two routes, from adult tick to larva (via the egg) or from rodent to larva, the density of *B. miyamotoi*-infected nymphs is less dependent on rodent density. Transmission, therefore, may be affected by various biotic and abiotic factors, which may vary temporally and geographically (Li et al. 2012; Perez et al. 2012).

Rodent species differ in their suitability as hosts for ticks and for *B. burgdorferi* s.l. (Kurtenbach et al. 1994; Matuschka et al. 1992; Nilsson and Lundqvist 1978). Tick burden also varies within rodent species, which may be due to differences in rodent characteristics (age, sex, immune status), microclimate or vegetation composition (Gassner et al. 2011; Randolph and Storey 1999). Climate and season are known to influence the behaviour of both ticks and rodents, and can also affect tick burdens on rodents (Estrada-Peña et al. 2013; Hubalek et al. 1994). The spatial aggregation of questing larvae in the environment may also contribute to the heterogeneity in larval tick burdens. The tick burden on white-footed mice (*Peromyscus leucopus*) was, however, consistent over time per individual mouse and was only slightly affected by this spatial aggregation (Devevey and Brisson 2012), suggesting a large effect of the rodent characteristics on rodent tick burdens. The interactions between ticks, rodents and *B. burgdorferi* s.l. are complex (Van Duijvendijk et al. 2015), and little is known about the ecology of *B. miyamotoi*. Most studies investigated only one or a few variables that potentially explain tick burdens on rodents, and hence the transmission dynamics of *Borrelia* spp. These variables may, however, also interact with each other. The relative contribution of each variable to *Borrelia* transmission can, therefore, not be compared, but is nevertheless important for understanding the ecology of the parasite communities.

The aim of this study was to quantify the infection rates of two *Borrelia* species, *B. burgdorferi* s.l. and *B. miyamotoi*, as well as their transmission rates in *I. ricinus* and in their rodent hosts. The study was conducted over several successive months during 2 years, so as to include the effects of spatiotemporal variation of the ecosystem on the factors being examined.

## Materials and methods

### Study sites

The study was conducted in a mixed forest near Wageningen, The Netherlands. Two plots of 55 × 55 m were selected with an inter-plot distance of 350 m. The undergrowth of plot A (51° 59′ 35.43" N, 5° 43′ 42.06" E) was dominated by wavy hairgrass (*Deschampsia flexuosa*), broad buckler-fern (*Dryopteris dilatata*) and blackberry (*Rubus fruticosus*), and that of plot B (51° 59′ 37.22" N, 5° 43′ 22.08" E) by broad buckler-fern (*D. dilatata*) and blackberry (*R. fruticosus*). Dominant tree species in both plots were scots pine (*Pinus sylvestris*) and silver birch (*Betula pendula*). The forest was part of a nature conservation area. Common mammal species present were bank vole (*Myodes glareolus*), wood mouse (*Apodemus sylvaticus*), common shrew (*Sorex araneus*), hare (*Lepus europaeus*), fox (*Vulpes vulpes*), badger (*Meles meles*) and roe deer (*Capreolus capreolus*). Bird populations consisted of a range of species common to mixed forests of north-western Europe.

### Rodent trapping

Rodents were trapped with Heslinga live traps provided with hay, grain, carrot, and a few mealworms. Traps were placed at 3-week intervals at 15:30 h and inspected the next day at 08.30 h, from May until November in 2013 and 2014 in both plots (10 trapping weeks each year). In 2013, plot B was started in July (7 trapping weeks). Traps were placed at 5 m inter-trap distance in grids of 12 × 12 traps in 2013 and 6 × 12 traps in 2014. Trapped rodents were identified to species level, sexed, weighed, and counted for ticks by searching the ears, snout, head, belly, legs, armpits, throat and tail. A small ear biopsy was collected from each captured male rodent with sterile scissors and tweezers during the first capture in 2013 and from males and females during each capture in 2014 (Sinsky and Piesman 1989). Ear biopsies were stored in 70% alcohol at – 20 ºC until further analysis. Rodents were individually marked by trimming their fur in a unique pattern. The fur was cut at nine locations on the body of a rodent (sides and back, at the front, middle or rear), allowing for 256 unique combinations (2^8^) per rodent species. A reference number and trap number were recorded for each rodent. A maximum of 15 male rodents per species was transported to the laboratory for the collection of feeding ticks (see also below). Captured shrews were released immediately. All experiments were approved by the ethical committee of Wageningen University (permit numbers 2013017 and 2014064).

### Rodent housing and tick collection

Trapped rodent males that were transported to the laboratory were housed individually in Makrolon type II cages placed over water-filled pans, with petroleum jelly on the edge to prevent ticks escaping. Cages were provided with standard rodent bedding and a tissue and toilet roll for shelter. Food and water were provided ad libitum. After 3 and 6 days, engorged ticks were collected from the water, dried on filter paper for 2 h and housed in ventilated 0.2-ml Eppendorf tubes at 20 ºC, 90–95% relative humidity and L14:D10 photoperiod. Engorged ticks were checked weekly and were stored at –20 ºC after they moulted to the next developmental stage until further analysis. In 2013, all emerged ticks were identified to species level. After 6 days, each rodent was released at the place of capture.

### Identification of *Borrelia burgdorferi* s.l. and *B. miyamotoi* infections

Ear biopsies and emerged nymphs were analysed individually as described by Sinsky and Piesman (1989). Briefly, nymphs were heated for 20 min at 99 °C in 100 μl 1 M ammonium hydroxide (NH_4_OH), after which they were centrifuged and heated for 20 min at 99 °C with open lids to evaporate the ammonia. The lysates were stored at 4 °C. DNA was extracted from the ear biopsies using the Qiagen DNeasy Blood & Tissue Kit (Jahfari et al. 2014). *Borrelia burgdorferi* s.l. DNA was detected using qPCR in the IQ Multiplex Powermix with a volume of 20 μl, containing iTaq DNA polymerase (Bio-Rad Laboratories, USA), 200 nM of each primer and 3 μl of template DNA (Heylen et al. 2013). Outer surface protein A gene (OspA) (forward primer: 5′-AAT ATT TAT TGG GAA TAG GTC TAA-3′; reverse primer: 5′-CTTTGT CTT TTT CTT TRC TTA CA-3′ and probe: 5′-Atto520-AAG CAA AAT GTT AGC AGC CTT GA-BHQ1-3′) and the *B. burgdorferi* s.l. flagellin gene (*flaB*) (forward primer: 5′-CAG AIA GAG GTT CTA TAC AIA TTG AIA TAG A-3′; reverse primer: 5′-GTG CAT TTG GTT AIA TTG YGC-3′ and probe: 5′-Atto425-CAA CTI ACA GAI GAA AXT AAI AGA ATT GCT GAI CA-Pho-3′, where X stands for an internal BHQ-1 quencher attached to thymine) were used as targets. The qPCR cycling program (using a light cycler 480 real-time PCR system; Hoffmann-La Roche, Switzerland) was performed using a two-step PCR program: Taq activation for 5 min at 95 °C followed by 60 cycles of 5 s at 94 °C and 35 s at 60 °C involving a single point measurement at 60 °C with corresponding filters, finishing with one cycle of 20 s at 37 °C. In the same qPCR, *B*. *miyamotoi* was detected with primers and probe based on the flagellin gene (*flaB*) for detection of the bacteria.

### Data analysis

Data were analysed with a generalized linear mixed model with a binomial distribution. Rodent identification number and trap number were used as random factors in each model. First, the effects of the main factors (year, rodent species, rodent sex, rodent weight, week, week^2^, rodent infection and plot) and all possible two-factor interactions between the main factors were analysed in four full models (Appendix).

The number of larvae counted in the field was used as dependent variable in the model to analyse larval tick burden on rodents. This model included all main factors and interactions. Data from ear biopsies were used as dependent variable in the model to analyse rodent infection rate. Rodent infection and the year × rodent sex interaction were excluded from this full model. The number of *B. burgdorferi* s.l. infected ticks out of the number of analysed ticks that were collected from the male rodents which were found *B. burgdorferi* s.l.-positive by PCR was used as dependent variable in the model to analyse rodent infectivity. Rodent sex and rodent infection were excluded from this full model. The number of moulted ticks out of the number of analysed ticks that were collected from the male rodents was used as dependent variable in the model to analyse larval moulting success. Rodent sex was excluded from this full model.

Second, each model was reduced by backwards elimination of the fixed factors, starting with the highest P-values until all factors were either significant, or were included in an interaction factor that was significant. Significance threshold was α = 0.05. All analyses were performed using SAS v.9.3 statistical software (SAS Institute, Cary, NC, USA).

## Results

### Rodent density

All trapped rodents were bank voles or wood mice (Table S1). In 2013, 78 bank voles and 51 wood mice were trapped during 2448 (1 plot × 144 traps × 7 nights + 1 plot × 144 traps × 10 nights) trap nights. In 2014, 443 bank voles and 287 wood mice were trapped during 1440 (2 plots × 72 traps × 10 nights) trap nights. Rodent density, analysed as the number of rodents trapped per trap night, was tenfold lower in 2013 (0.053, 129/2448) than in 2014 (0.507, 730/1440).

### Tick burden

In total, 3157 larvae were counted in the field on the trapped rodents (Table S1). Mean larval tick burden was 7.02 ± 0.87 in 2013 and 3.08 ± 0.22 in 2014. Larvae were highly aggregated among rodents; in 2013, 20% of the rodents fed 62.3% of the larvae, and in 2014, 19% of the rodents fed 73.4% of the larvae. In 2013, 1128 larvae were collected in the laboratory from male rodents, from which 1 086 were identified to species level after they moulted into nymphs. One of these was *I. trianguliceps*, all others were *I. ricinus.* In 2014, 1410 larvae were collected in the laboratory from male rodents, which were not identified to species level. In 2013, 33 nymphs were collected in the laboratory from male rodents, from which 17 were identified to species level after they had moulted into adults. Two of these were *I. trianguliceps*, all others were *I. ricinus.* In 2014, 32 nymphs were collected in the laboratory from male rodents, which were not identified to species level. No adult ticks were collected from the water basins, even though four (unidentified) adult ticks were observed feeding on the rodents during trapping.

**Fig. 1:**
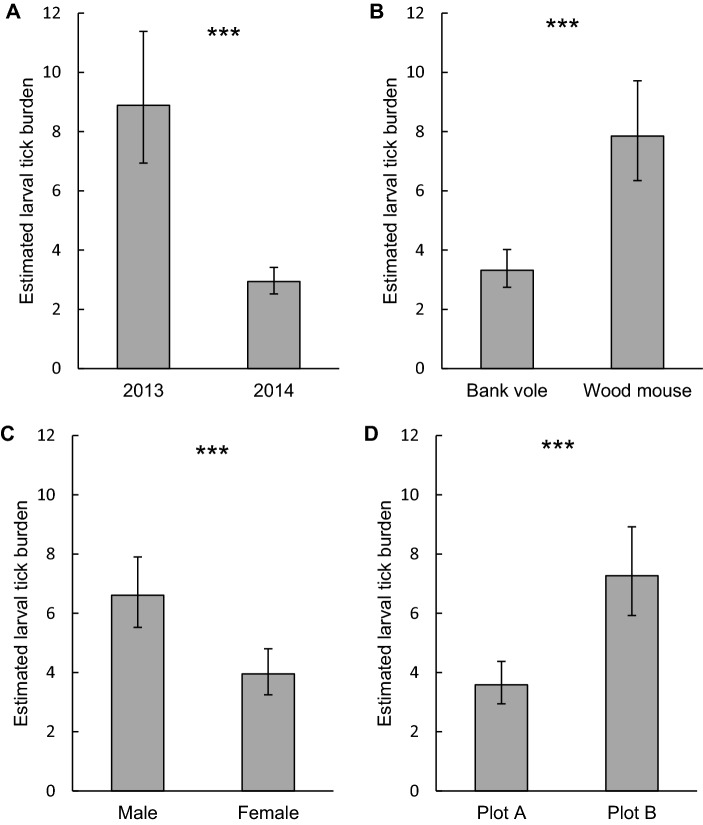
Estimated mean larval tick burden on rodents in week 32 per **A** year, **B** rodent species, **C** rodent sex, and **D** plot. Error bars represent 95% confidence intervals. Significant differences between means are indicated with ***P < 0.001 (generalized linear mixed model)

**Fig. 2:**
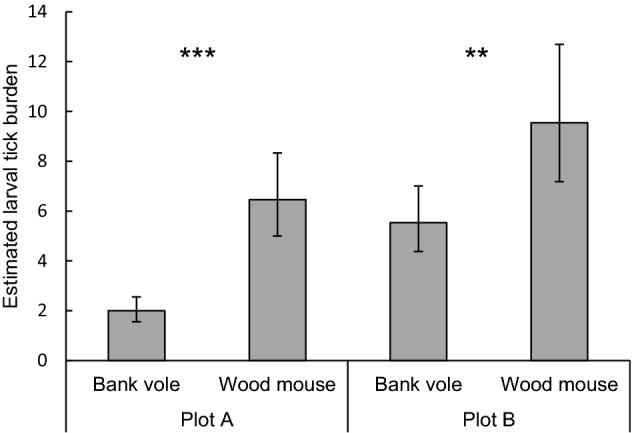
Estimated mean larval tick burden on rodents in week 32 per rodent species per plot. Error bars represent 95% confidence intervals. Significant differences between means are indicated with **P < 0.01, ***P < 0.001 (generalized linear mixed model)

**Fig. 3:**
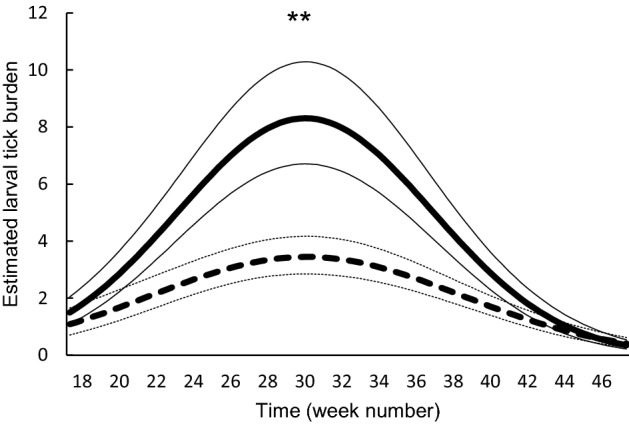
Estimated larval tick burden on wood mice (solid line) and bank voles (dashed line) over time. Thin lines represent upper and lower bounds of the 95% confidence intervals. Significant differences between the solid and dashed lines are indicated with **P < 0.01 (generalized linear mixed model)

**Fig. 4:**
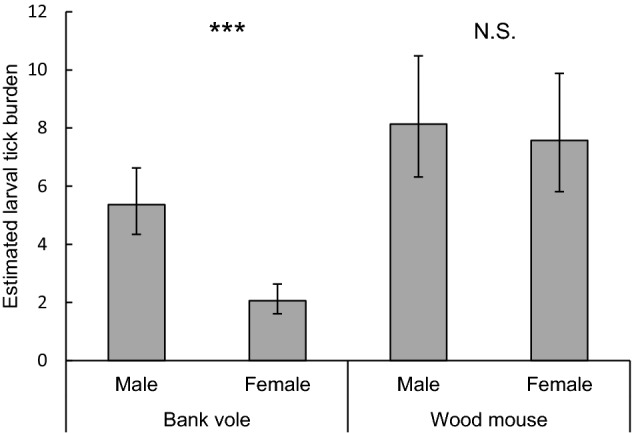
Estimated mean larval tick burden on rodents in week 32 per rodent sex per rodent species. Error bars represent 95% confidence intervals. Significant differences between means are indicated with ***P < 0.001; N.S.: P > 0.05 (generalized linear mixed model)

Larval tick burden differed between years and was significantly higher in 2013 (8.9, 95% confidence interval [95% CI] 6.9–11.4) than in 2014 (2.9, 95% CI 2.5–3.4; P < 0.0001; Fig. 1A). Larval tick burden differed between rodent species and was lower on bank voles (3.3, 95% CI 2.7–4.0) than on wood mice (7.9, 95% CI 6.3–9.7; P < 0.0001; Fig. 1B). The difference in larval tick burden between rodent species varied between plots (P = 0.0021); on average, bank voles had lower larval tick burdens than wood mice, but the effect of rodent species on larval tick burden was larger in plot A (P < 0.0001) than in plot B (P < 0.0011; Fig. 2). The difference in larval tick burden between rodent species varied over time (P = 0.0039; Fig. 3); the difference was larger in summer than in spring and autumn. Males had higher larval tick burdens (6.6, 95% CI 5.5–7.9) than females (3.9, 95% CI 3.2–4.8; P < 0.0001; Fig. 1C). The difference in larval tick burden between males and females varied between rodent species (P < 0.0001); in bank voles, males had higher larval tick burdens than females (P < 0.0001), whereas sex did not affect larval tick burden on wood mice (P = 0.64; Fig. 4). Larval tick burden differed between plots, and was lower in plot A (3.6, 95% CI 2.9–4.4) than in plot B (7.3, 95% CI 5.9–8.9; P < 0.0001; Fig. 1D). The difference in larval tick burden between plots varied over time (P = 0.017). Rodents having a higher body weight had larger larval tick burdens than rodents weighing less (P < 0.0001; Figure S1). With each increasing gram of body weight, larval tick burden was multiplied by 1.06. Larval tick burden varied over time (P < 0.0001; Fig. 3).

**Fig. 5:**
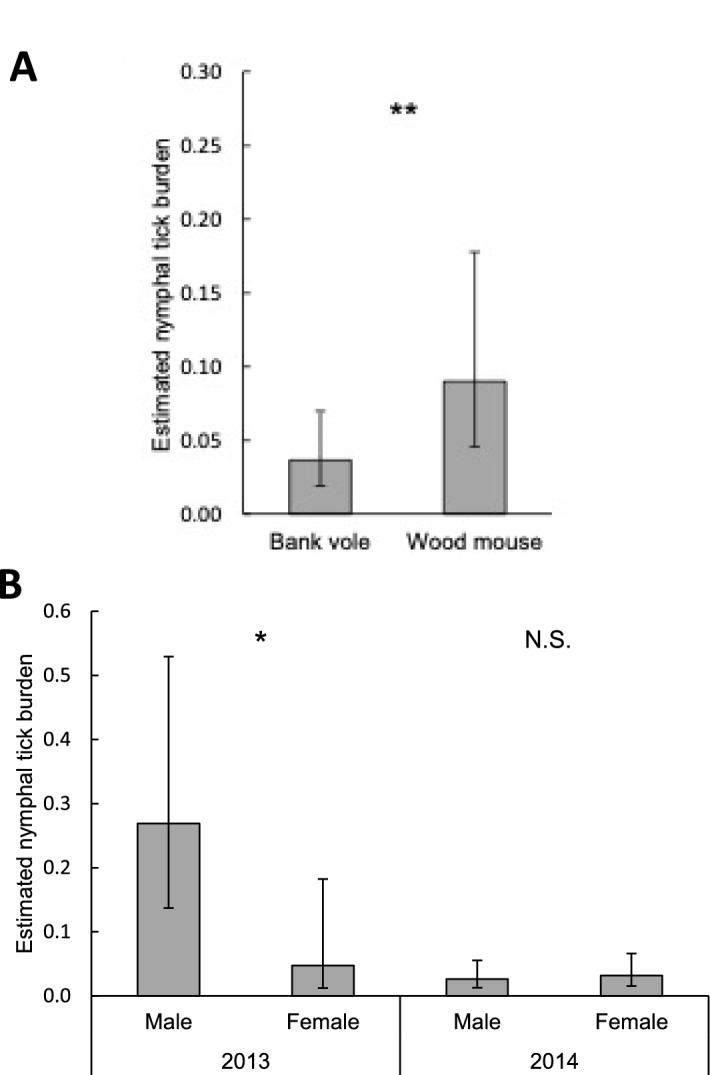
Estimated mean nymphal tick burden on male and female rodents in week 32 per **A** rodent species and **B** year. Error bars represent 95% confidence intervals. Significant differences between means are indicated with *P < 0.05, **P < 0.01; N.S.: P > 0.05 (generalized linear mixed model)

Bank voles had lower nymphal tick burdens (0.04, 95% CI 0.02–0.07) than wood mice (0.09, 95% CI 0.05–0.18; P = 0.0081; Fig. 5A). In 2013, males had higher nymphal tick burdens than females (P = 0.015), whereas in 2014 the nymphal tick burden did not differ between males and females (P = 0.66; Fig. 5B).

Nymphal tick burden was higher in 2013 (0.11, 95% CI 0.05–0.25) than in 2014 (0.03, 95% CI 0.02–0.05; P = 0.0015; Figure S2). Rodents having a higher body weight had larger nymphal tick burdens than rodents weighing less (P = 0.0004; Figure S3). With each gram of body weight, larval tick burden was multiplied by 1.19.

### Rodent infection rate

In 2013, 60 ear biopsies were analysed for infection, of which 21 (35%) were infected with *B. burgdorferi* s.l. and three (5%) were infected with *B. miyamotoi* (Table S2). In 2014, 705 ear biopsies were analysed for infection, from which 100 (14%) were infected with *B. burgdorferi* s.l. and eight (1%) were infected with *B. miyamotoi*.

**Fig. 6:**
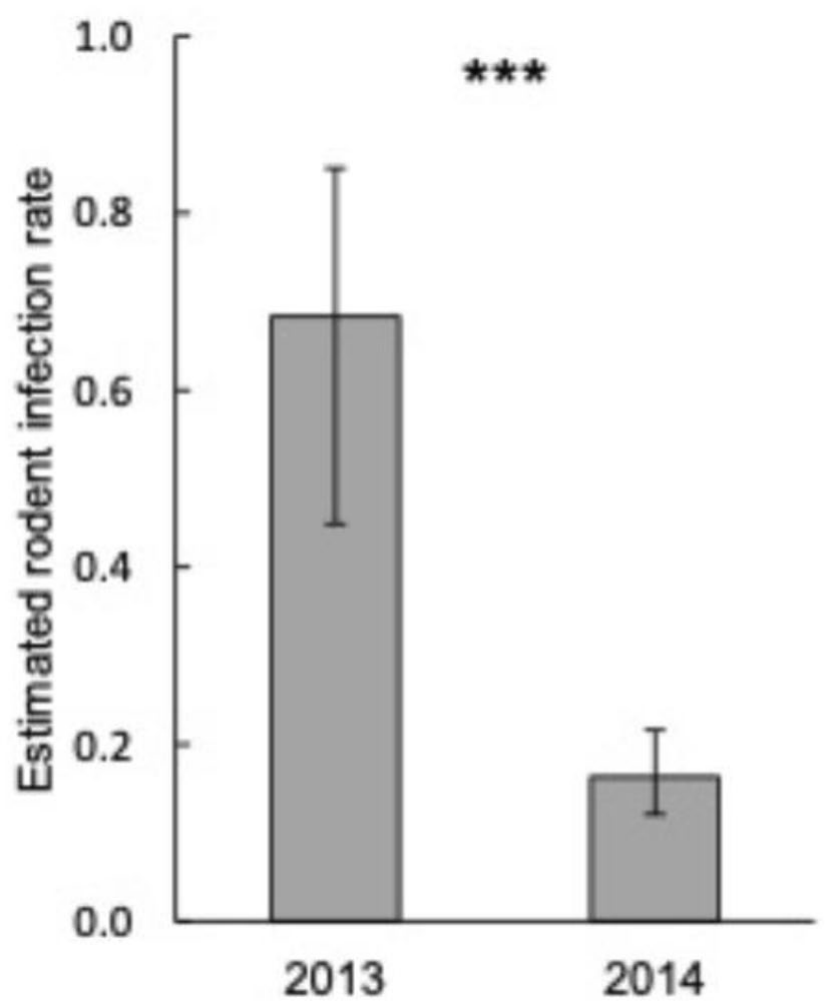
Estimated mean infection rate of rodents with *Borrelia burgdorferi* s.l. in week 32 per year. Error bars represent 95% confidence intervals. Significant differences between the means are indicated with ***P < 0.001 (generalized linear mixed model)

**Fig. 7:**
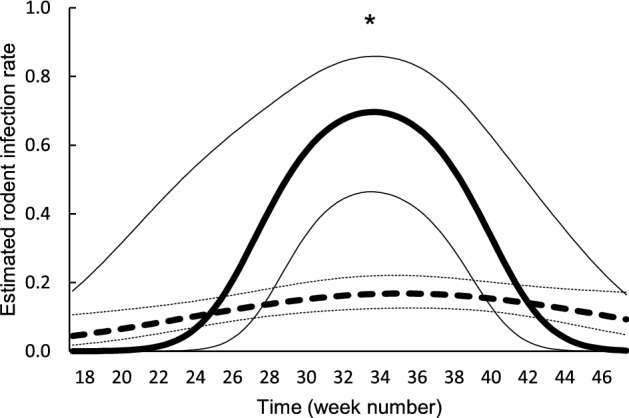
Estimated rodent infection rate in 2013 (solid line) and 2014 (dashed line) over time. Thin lines represent upper and lower bounds of the 95% confidence intervals. Significant differences between the solid and dashed lines are indicated with *P < 0.05 (generalized linear mixed model)

Rodent infection rate with *B. burgdorferi* s.l. was higher in 2013 (0.68, 95% CI 0.45–0.85) than in 2014 (0.16, 95% CI 0.12–0.22; P < 0.0001; Fig. 6). The difference in rodent infection rate between years varied over time (P = 0.0190; Fig. 7). There was a shorter, but higher peak in 2013 than in 2014. Rodent infection rate also varied over time (P = 0.0034; Fig. 7), peaking in summer. Rodents weighing more had a higher infection rate than those weighing less (P = 0.0011; Figure S4). For each increasing gram of body weight, rodent infection rate increased by a factor 1.09. The effect of rodent sex on rodent infection rate varied over time (P = 0.022; Figure S5). Rodent infection rate was highest in week 32–36 for both sexes.

### Rodent infectivity

In 2013, 496 larvae from *B. burgdorferi* s.l.-infected rodents were analysed for infection, of which 228 (46%) were infected with *B. burgdorferi* s.l. (Table S2). In 2013, 22 larvae from *B. miyamotoi*-infected rodents were analysed for infection, of which seven (32%) were infected with *B. miyamotoi*. In 2014, 246 larvae from *B. burgdorferi* s.l.-infected rodents were analysed for infection, of which 94 (38%) were infected with *B. burgdorferi* s.l. In 2014, eight larvae from *B. miyamotoi*-infected rodents were analysed for infection, of which three (38%) were infected with *B. miyamotoi*. For *B. burgdorferi* s.l., estimated rodent infectivity was 0.38 (95% CI 0.30–0.47) and was not affected by any of the main factors or two-factor interactions P > 0.05 (Table S1).

### Moulting success of larvae

In 2013, 1128 engorged larvae were collected from the male rodents in the laboratory, of which 1086 (96%) moulted into nymphs (Table S2). In 2014, 1410 engorged larvae were collected from the male rodents in the laboratory, of which 1349 (96%) moulted into nymphs. Moulting success was lower for plot A (0.91, 95% CI 0.88–0.94) than for plot B (0.95, 95% CI 0.93–0.96; P = 0.044; Figure S6). Moulting success did not differ between rodent species (P = 0.057). The effect of rodent species was affected by year (P = 0.018); in 2013, moulting success was not affected by rodent species (P = 0.74) but in 2014, moulting success was higher for larvae that fed on bank voles compared to larvae that fed on wood mice (P = 0.0016; Figure S7). Moulting success of larvae varied over time (P < 0.0001).

### Contribution of rodents to the indirect estimation of the density of infected nymphs

The calculated relative numbers of infected questing nymphs per year, rodent species, plot and rodent sex are shown in Table S2. The relative number of *B. burgdorferi* s.l.-infected questing nymphs that emerged from the larvae that fed on the rodents was 0.058 for 2013 and 0.081 for 2014. The relative number of *B. miyamotoi*-infected questing nymphs that emerged from the larvae that fed on the rodents was the same for 2013 and 2014 (0.00006).

## Discussion

The most common rodent species in The Netherlands, bank voles and wood mice, were the only rodent species trapped in this study (de Boer et al. 1993; Gassner et al. 2008).

Rodent density was about one order of magnitude lower in 2013 than in 2014. The contribution of the 2013 rodent population to the density of *B. burgdorferi* s.l.-infected nymphs was, however, only a little less than that of the 2014 population. The contribution of these rodents to the density of *B. miyamotoi*-infected nymphs did not differ between years.

The total number of feeding larvae was higher, whereas the estimated larval and nymphal tick burdens per rodent were lower in 2014 than in 2013. These lower tick burdens caused a lower rodent infection rate with *B. burgdorferi* s.l. in 2014. As a result, the contribution of the 2014 population to the density of *B. burgdorferi* s.l.-infected nymphs increased only slightly. The lower mean larval tick burden in 2014 was probably due to a dilution effect caused by the high rodent density compared to that of 2013 (Kiffner et al. 2011; Krasnov et al. 2007; Rosa et al. 2007). Rodent density is largely affected by food availability, which also affects home range size (Stradiotto et al. 2009). A large home range size of rodents can result in more tick encounters and therefore, a higher tick burden (Devevey and Brisson 2012; Sonenshine and Stout 1968). The contribution of the rodent populations to the density of *B. miyamotoi*-infected nymphs, however, did not differ between the years, supporting the importance of transovarial transmission for this spirochaete (Hauck et al. 2020; Krause et al. 2015).

Our data showed that larval and nymphal tick burdens were higher on wood mice than on bank voles, which is supported by previous studies in different ecological settings (Gassner et al. 2013; Mysterud et al. 2015; Tälleklint and Jaenson 1997). In addition, more larvae were found on male rodents than on female rodents and on rodents with a large body weight, which are in general older than smaller ones, which is also supported by previous studies from different ecological settings (Devevey and Brisson 2012; Nilsson and Lundqvist 1978; Sonenshine and Stout 1968; Tälleklint and Jaenson 1997). The higher tick burdens on male compared to female rodents may be the effect of larger home ranges of males (Stradiotto et al. 2009) and therefore tick encounter rates.

Larval and nymphal tick burdens were higher in 2013 compared to 2014. Considering Lyme borreliosis risk, only infected rodents can infect feeding larvae. Rodents can become infected with *B. burgdorferi* s.l. through the bite of an infected larva or nymph (Radolf et al. 2012; Van Duijvendijk et al. 2016). A high tick burden on rodents, therefore, results in a high exposure to the spirochaetes. As a result of this higher tick burden, rodent infection rate with *B. burgdorferi* s.l. was higher in 2013 compared to 2014. The lower rodent infection rate in 2014 contributed to a small increase in the density of *B. burgdorferi* s.l.-infected nymphs fed by the 2014 rodent population compared to the 2013 rodent population. Variation in rodent infection rate between years was also found by Kurtenbach et al. (1995) and Krawczyk et al (2020) and is most likely caused by the annual variations in the ratio of rodent/questing tick density, with a dilution effect at high rodent densities, leading to lower *B. burgdorferi* s.l. infections in rodents. In our study, rodent infection rate peaked during summer, which would increase the infection rate in larvae that feed on the rodent population. An increase in rodent infection rate in the spring was also found by Kurtenbach et al. (1995). These authors did not, however, find a decrease in the infection rate of feeding larvae in the autumn and speculated that this difference may have been caused by a difference in longevity of the rodents. In our study, the rodent infection rate showed a similar peak over time to that of the larval tick burden. Larval tick burden is likely to be affected by larval questing activity. Likewise, tick questing activity affected the infection rate of white footed mice (Hofmeister et al. 1999). We did not find a difference in infection rate between rodent species, which is in contradiction to the results of Kybicova et al. (2008), Humair et al. (1999) and Krawczyk et al. (2020), who found a higher infection rate in bank voles than in wood mice. It is unclear what caused this difference. Gassner et al (2013), however, reported variable infection rates between bank voles and wood mice, collected from three geographically different areas. Our molecular analysis did not distinguish between the *B. burgdorferi* s.l. genospecies, but previous research showed that rodents and larvae collected from rodents are mainly infected with *B. afzelii* (Hanincova et al. 2003; Humair et al. 1999; Kybicova et al. 2008), which can cause skin problems in humans (Stanek et al. 2012; Strle and Stanek 2009).

For the infected rodents to have an effect on the density of infected nymphs, the spirochaetes should also be transmitted to feeding larvae. Rodent infectivity was 0.43 based on the raw data, and estimated rodent infectivity was 0.38 when corrected for trap number and rodent number. These values are in line with the findings of Burri et al. (2014). Rodent infectivity was not affected by any of the measured variables. This is in contrast with the results of Perez et al. (2012) and Humair et al. (1999), who found a higher infectivity of bank voles than of wood mice. Infected rodents do not lose their infection, but the infectivity varies between individual rodents and was enhanced by successive infestations with larval ticks (Gern et al. 1994).

Overall, moulting success of larvae was always > 90% and was not affected by rodent species. This is in line with the findings of Tälleklint and Jaenson (1997), but in contradiction to the results of Humair et al. (1999), who found a higher moulting rate of ticks that fed on wood mice (33.3%) compared to bank voles (6.2%). We found that the effect of rodent species varied between years. Dizij and Kurtenbach (1995) showed that moulting success of larvae that fed on bank voles was reduced with successive tick infestations, whereas this was not found for larvae feeding on wood mice. Years with high tick burdens could, therefore, result in reduced moulting success for ticks that fed on bank voles, possibly caused by immunity to tick antigens. We found, however, that moulting success was significantly decreased in bank voles in 2014, when tick burdens were low.

Understanding the factors that cause the aggregation of ticks on rodents helps to understand the transmission dynamics of *B. afzelii* and to identify the 20% of the rodents that contribute to 80% of the *B. afzelii* transmission cycle (Lloyd-Smith et al. 2005; Stein 2011; Woolhouse et al., 1997). Larval density, and not rodent density was found to be the best explanatory variable for nymphal density in the following year (Rosa et al. 2007).

Larval tick burdens on rodents show high temporal variation within and between years. A peak in rodent density (e.g., after a mast year) results in reduced tick burdens on rodents and a reduced rodent infection rate.

We conclude that years with high rodent densities do not lead to an increase in the contribution of the rodent population to the density of *B. burgdorferi* s.l.-infected nymphs and *B. miyamotoi*-infected nymphs. The risks for Lyme borreliosis and HTRF are, therefore, not directly affected by a temporal variation in rodent density. The strong seasonal variation in tick burden on rodents as well as the variation in rodent infection rates, however, cause large differences in *Borrelia* infection risk and should be considered in public health campaigns aimed at the prevention of tick-borne disease.

### Supplementary Information

Below is the link to the electronic supplementary material.

Supplementary file1 (DOCX 788 kb)

## Data Availability

The datasets generated during and/or analysed during the current study are available from the corresponding author on reasonable request.

## Appendix

See Table 1.

**Table 1:** Main factors and two-factor interactions that were included in the generalized linear mixed models for analysing the effects on larval tick burden, rodent infection, rodent infectivity, and tick moulting success and their corresponding F- and P-values

	Larval tick burden		Nymphal tick burden		Rodent infection rate		Rodent infectivity		Larval moulting success	
	F	P	F	P	F	P	F	P	F	P
Y	70.89	< 0.0001	10.14	0.0015	22.11	< 0.0001		–	0.03	0.8748
Sp	46.56	< 0.0001	7.11	0.0081		–		–	3.76	0.0567
Sx	26.40	< 0.0001	3.55	0.0600	0.48	0.4879		X		X
W	45.70	< 0.0001	12.67	0.0004	11.20	0.0011		–		–
T	49.95	< 0.0001	0.04	0.8356	1.65	0.2017		–	37.69	< 0.0001
T^2^	222.73	< 0.0001	0.18	0.6712	8.96	0.0034		–		–
RI		–		–		X		X		–
P	31.98	< 0.0001	0.04	0.8501		–		–	4.22	0.0437
Y*Sp		–		–		–		–	5.87	0.0182
Y*Sx		–	5.43	0.0203		X		X		X
Y*W		–		–		–		–		–
Y*T		–		–	0.58	0.4485		–		–
Y*T^2^		–		–	5.67	0.0190		–		–
Y*P		–		–		–		–		–
Y*RI		–		–		X		X		–
Sp*Sx	19.77	< 0.0001		–		–		X		X
Sp*W		–		–		–		–		–
Sp*T	2.33	0.1272		–		–		–		–
Sp*T^2^	8.40	0.0039		–		–		–		–
Sp*P Sp*	9.56	0.0021		–		–		–		–
Sp*RI		–		–		X		X		–
Sx*W		–		–		–		X		X
Sx*T		–		–	5.43	0.0218		X		X
Sx*T^2^		–		–	7.15	0.0087		X		X
Sx*RI		–		–		X		X		X
Sx*P		–		–		–		X		X
W*T		–	0.00	0.9808		–		–		–
W*T^2^		–	6.90	0.0090		–		–		–
W*RI		–		–		X		X		–
W*P		–		–		–		–		–
T*RI		–		–		X		–		–
T^2^*P		–		–		–		–		–
T^2^*RI		–		–		X		–		–
T*P	5.68	0.0174	4.18	0.0414		–		–		–
RI*P		–		–		X		–		–

## References

[CR1] Barbour AG, Bunikis J, Travinsky B, Hoen AG, Diuk-Wasser MA, Fish D, Tsao JI (2009). Niche partitioning of *Borrelia burgdorferi* and *Borrelia miyamotoi* in the same tick vector and mammalian reservoir species. Am J Trop Med Hyg.

[CR2] Burri C, Schumann O, Schumann C, Gern L (2014). Are *Apodemus* spp. mice and *Myodes glareolus* reservoirs for *Borrelia miyamotoi*, Candidatus *Neoehrlichia mikurensis*, *Rickettsia helvetica*, *R. monacensis* and *Anaplasma phagocytophilum*?. Ticks Tick-Borne Dis.

[CR3] de Boer R, Hovius KE, Nohlmans MK, Gray JS (1993). The woodmouse (*Apodemus sylvaticus*) as a reservoir of tick-transmitted spirochetes (*Borrelia burgdorferi*) in The Netherlands. Zentralbl Bakteriol.

[CR4] Devevey G, Brisson D (2012). The effect of spatial heterogenity on the aggregation of ticks on white-footed mice. Parasitology.

[CR5] Dizij A, Kurtenbach K (1995). *Clethrionomys glareolus*, but not *Apodemus flavicollis* acquires resistance to *Ixodes ricinus* L., the main European vector of *Borrelia burgdorferi*. Parasite Immunol.

[CR6] Estrada-Peña A, Gray JS, Kahl O, Lane RS, Nijhof AM (2013). Research on the ecology of ticks and tick-borne pathogens–methodological principles and caveats. Front Cell Infect Microbiol.

[CR7] Gassner F, Takken W, Lombaers-van der Plas C, Kastelein P, Hoetmer AJ, Holdinga M, van Overbeek LS (2013). Rodent species as natural reservoirs of *Borrelia burgdorferi* sensu lato in different habitats of *Ixodes ricinus* in The Netherlands. Ticks Tick-Borne Dis.

[CR8] Gassner F, van Vliet AJ, Burgers SL, Jacobs F, Verbaarschot P, Hovius EK, Mulder S, Verhulst NO, van Overbeek LS, Takken W (2011). Geographic and temporal variations in population dynamics of *Ixodes ricinus* and associated *Borrelia* infections in The Netherlands. Vector Borne Zoonotic Dis.

[CR9] Gassner F, Verbaarschot P, Smallegange RC, Spitzen J, Van Wieren SE, Takken W (2008). Variations in *Ixodes ricinus* density and *Borrelia* infections associated with cattle introduced into a woodland in The Netherlands. Appl Environ Microbiol.

[CR10] Gern L, Siegenthaler M, Hu CM, Leuba-Garcia S, Humair PF, Moret J (1994). *Borrelia burgdorferi* in rodents (*Apodemus flavicollis* and *A. sylvaticus*): duration and enhancement of infectivity for *Ixodes ricinus* ticks. Eur J Epidemiol.

[CR11] Hanincova K, Schäfer SM, Etti S, Sewell HS, Taragelová V, Ziak D, Labuda M, Kurtenbach K (2003). Association of *Borrelia afzelii* with rodents in Europe. Parasitology.

[CR12] Harrison A, Bennett NC (2012). The importance of the aggregation of ticks on small mammal hosts for the establishment and persistence of tick-borne pathogens: an investigation using the R 0 model. Parasitology.

[CR13] Hauck D, Jordan D, Springer A, Schunack B, Pachnicke S, Fingerle V, Strube C (2020). Transovarial transmission of *Borrelia* spp., *Rickettsia* spp. and *Anaplasma phagocytophilum* in *Ixodes ricinus* under field conditions extrapolated from DNA detection in questing larvae. Parasit Vectors.

[CR14] Heylen D, Tijsse E, Fonville M, Matthysen E, Sprong H (2013). Transmission dynamics of *Borrelia burgdorferi* s.l. in a bird tick community. Environ Microbiol.

[CR16] Hofmeister EK, Ellis BA, Glass GE, Childs JE (1999). Longitudinal study of infection with *Borrelia burgdorferi* in a population of *Peromyscus leucopus* at a Lyme disease-enzootic site in Maryland. Am J Trop Med Hyg.

[CR15] Hofmeester TR, Coipan EC, van Wieren SE, Prins HHT, Takken W, Sprong H (2016). Few vertebrate species dominate the *Borrelia burgdorferi* s.l. life cycle. Environ Res Lett.

[CR17] Hubalek Z, Halouzka J, Juricova Z, Svobodova S (1994). Seasonal distribution of *Borreliae* in *Ixodes ricinus* ticks. Zentralbl Bakteriol.

[CR18] Humair PF, Rais O, Gern L (1999). Transmission of *Borrelia afzelii* from *Apodemus* mice and *Clethrionomys* voles to *Ixodes ricinus* ticks: differential transmission pattern and overwintering maintenance. Parasitology.

[CR19] Jahfari S, Coipan EC, Fonville M, van Leeuwen AD, Hengeveld P, Heylen D, Heyman P, van Maanen C, Butler CM, Foldvari G, Szekeres S, van Duijvendijk G, Tack W, Rijks JM, van der Giessen J, Takken W, van Wieren SE, Takumi K, Sprong H (2014). Circulation of four *Anaplasma phagocytophilum* ecotypes in Europe. Parasit Vector.

[CR20] Kiewra D, Stańczak J, Richter M (2014). *Ixodes ricinus* ticks (Acari, Ixodidae) as a vector of *Borrelia burgdorferi* sensu lato and *Borrelia miyamotoi* in Lower Silesia, Poland—preliminary study. Ticks Tick-Borne Dis.

[CR21] Kiffner C, Vor T, Hagedorn P, Niedrig M, Rühe F (2011). Factors affecting patterns of tick parasitism on forest rodents in tick-borne encephalitis risk areas, Germany. Parasitol Res.

[CR22] Kjelland V, Rollum R, Korslund L, Slettan A, Tveitnes D (2015). *Borrelia miyamoto*i is widespread in *Ixodes ricinus* ticks in southern Norway. Ticks Tick-Borne Dis.

[CR23] Krasnov BR, Stanko M, Morand S (2007). Host community structure and infestation by Ixodid ticks: repeatability, dilution effect and ecological specialization. Oecologia.

[CR24] Krause PJ, Fish D, Narasimhan S, Barbour AG (2015). *Borrelia miyamotoi* infection in nature and in humans. Clin Microbiol Infect.

[CR25] Krawczyk AI, van Duijvendijk GLA, Swart A, Heylen D, Jaarsma RI, Jacobs FHH, Fonville M, Sprong H, Takken W (2020). Effect of rodent density on tick and tick-borne pathogen populations: consequences for infectious disease risk. Parasit Vector.

[CR26] Kurtenbach K, Dizij A, Seitz HM, Margos G, Moter SE, Kramer MD, Wallich R, Schaible UE, Simon MM (1994). Differential immune-responses to *Borrelia burgdorferi* in European wild rodent species influence spirochete transmission to *Ixodes ricinus* L (Acari, Ixodidae). Infect Immun.

[CR27] Kurtenbach K, Kampen H, Dizij A, Arndt S, Seitz HM, Schaible UE, Simon MM (1995). Infestation of rodents with larval *Ixodes ricinus* (Acari: Ixodidae) is an important factor in the transmission cycle of *Borrelia burgdorferi* s.l. in German woodlands. J Med Entomol.

[CR28] Kybicova K, Kurzova Z, Hulinska D (2008). Molecular and serological evidence of *Borrelia burgdorferi* sensu lato in wild rodents in the Czech Republic. Vector-Borne Zoonot Dis.

[CR29] Li S, Heyman P, Cochez C, Simons L, Vanwambeke SO (2012). A multi-level analysis of the relationship between environmental factors and questing *Ixodes ricinus* dynamics in Belgium. Parasit Vector.

[CR30] Lloyd-Smith JO, Schreiber SJ, Kopp PE, Getz WM (2005). Superspreading and the effect of individual variation on disease emergence. Nature.

[CR31] Matuschka FR, Fischer P, Heiler M, Richter D, Spielman A (1992). Capacity of European animals as reservoir hosts for the Lyme disease spirochete. J Infect Dis.

[CR32] Mysterud A, Byrkjeland R, Qviller L, Viljugrein H (2015). The generalist tick *Ixodes ricinus* and the specialist tick *Ixodes trianguliceps* on shrews and rodents in a northern forest ecosystem—a role of body size even among small hosts. Parasit Vector.

[CR33] Nilsson A, Lundqvist L (1978). Host selection and movements of *Ixodes ricinus* (Acari) larvae on small mammals. Oikos.

[CR34] Perez D, Kneubuhler Y, Rais O, Gern L (2012). Seasonality of *Ixodes ricinus* ticks on vegetation and on rodents and *Borrelia burgdorferi* sensu lato genospecies diversity in two Lyme borreliosis-endemic areas in Switzerland. Vector-Borne Zoonotic Dis.

[CR35] Perkins SE, Cattadori IM, Tagliapietra V, Rizzoli AP, Hudson PJ (2003). Empirical evidence for key hosts in persistence of a tick-borne disease. Int J Parasitol.

[CR36] Radolf JD, Caimano MJ, Stevenson B, Hu LT (2012). Of ticks, mice and men: understanding the dual-host lifestyle of Lyme disease spirochaetes. Nat Rev Microbiol.

[CR37] Randolph SE, Storey K (1999). Impact of microclimate on immature tick-rodent host interactions (Acari: Ixodidae): implications for parasite transmission. J Med Entomol.

[CR38] Rosa R, Pugliese A, Ghosh M, Perkins SE, Rizzoli A (2007). Temporal variation of *Ixodes ricinus* intensity on the rodent host *Apodemus flavicollis* in relation to local climate and host dynamics. Vector-Borne Zoonotic Dis.

[CR39] Scoles GA, Papero M, Beati L, Fish D (2001). A relapsing fever group spirochete transmitted by *Ixodes scapularis* ticks. Vector-Borne Zoonotic Dis.

[CR40] Sinsky R, Piesman J (1989). Ear punch biopsy method for detection and isolation of *Borrelia burgdorferi* from rodents. J Clin Microbiol.

[CR41] Sonenshine DE, Stout J (1968). Tick burdens in relation to spacing and range of hosts in *Dermacentor variabilis*. J Med Entomol.

[CR42] Stanek G, Wormser GP, Gray J, Strle F (2012). Lyme borreliosis. Lancet.

[CR43] Stein RA (2011). Super-spreaders in infectious diseases. Int J Infect Dis.

[CR44] Stradiotto A, Cagnacci F, Delahay R, Tioli S, Nieder L, Rizzoli A (2009). Spatial organization of the yellow-necked mouse: effects of density and resource availability. J Mammal.

[CR45] Strle F, Stanek G (2009). Clinical manifestations and diagnosis of lyme borreliosis. Curr Probl Dermatol.

[CR46] Tälleklint L, Jaenson TG (1997). Infestation of mammals by *Ixodes ricinus* ticks (Acari: Ixodidae) in south-central Sweden. Exp Appl Acarol.

[CR48] van Duijvendijk G, Sprong H, Takken W (2015). Multi-trophic interactions driving the transmission cycle of *Borrelia afzelii* between *Ixodes ricinus* and rodents: a review. Parasit Vector.

[CR47] van Duijvendijk G, Coipan C, Wagemakers A, Fonville M, Ersoz J, Oei A, Foldvari G, Hovius J, Takken W, Sprong H (2016). Larvae of *Ixodes ricinus* transmit *Borrelia afzelii* and *B. miyamotoi* to vertebrate hosts. Parasit Vector.

[CR49] Woolhouse MEJ, Dye C, Etard JF, Smith T, Charlwood JD, Garnett GP, Hagan P, Hii JLK, Ndhlovu PD, Quinnell RJ, Watts CH, Chandiwana SK, Anderson RM (1997). Heterogeneities in the transmission of infectious agents: Implications for the design of control programs. Proc Natl Acad Sci USA.

