# Supplementary material for: Seasonal dynamics of tick burden and associated Borrelia burgdorferi s.l. and Borrelia miyamotoi infections in rodents in a Dutch forest ecosystem
Source: Exp Appl Acarol. 2022 Jul 16;87(2-3):235–51. doi: 10.1007/s10493-022-00720-z (PMC9424142; doi:10.1007/s10493-022-00720-z)
Supplement: Supplementary file 1 — Supplementary file1 (DOCX 788 kb) [file 10493_2022_720_MOESM1_ESM.docx]

**SUPPLEMENTARY INFORMATION**

**Figure S1** - Estimated larval tick burden on rodents in week 32 by rodent weight. Thin lines represent upper and lower bounds of the 95% confidence intervals. Significant differences are indicated with ***P<0.001 (generalized linear mixed model).

**Figure S2** - Estimated mean nymphal tick burden on rodents in week 32 per rodent sex per year. Error bars represent 95% confidence intervals. Significant differences between means are indicated with *P<0.05 (generalized linear mixed model).

**Figure S3** - Estimated nymphal tick burden on rodents in week 32 by rodent weight. Thin lines represent upper and lower bounds of the 95% confidence intervals. Significant differences (between low-weight and high-weight groups) are indicated with ***P<0.001 (generalized linear mixed model).

**Figure S4** - Estimated rodent infection rate in week 32 by rodent weight. Thin lines represent upper and lower bounds of the 95% confidence intervals. Significant differences are indicated with **P<0.01 (generalized linear mixed model).

**Figure S5** - Estimated rodent infection rate of male (solid line) and female (dashed line) rodents over time. Thin lines represent upper and lower bounds of the 95% confidence intervals. Significant indications are indicated with **P<0.01 (generalized linear mixed model).

**Figure S6** - Estimated moulting success of larvae per plot. Error bars represent 95% confidence intervals. Significant indications are indicated with *P<0.05 (generalized linear mixed model).

**Figure S7** - Estimated moulting success of larvae per rodent species per year. Error bars represent 95% confidence intervals. Significant indications are indicated with *P<0.05 (generalized linear mixed model).

**Table S1**. Larval and nymphal tick burdens of bank voles and wood mice in 2013 and 2014 per plot and rodent sex.

|  |  | Larvae | | |  | | | Nymphs | | |
| --- | --- | --- | --- | --- | --- | --- | --- | --- | --- | --- |
|  | Relative rodent density  (Analysed rodents)* | Infested rodents (%) | Total | Mean burden  ± SE | |  | Infested rodents (%) | | Total | Mean burden  ± SE |
| **2013** | 0.053 (129) | **105 (78.9)** | **905** | **7.02 ± 0.87** | |  | **17 (12.8)** | | **31** | **0.24 ± 0.07** |
| **Bank voles** | 0.032 (78) | **60 (74.1)** | **394** | **5.05 ± 0.70** | |  | **8 (9.9)** | | **9** | **0.12 ± 0.04** |
| *Plot A* | 0.031 (45) | 32 (66.7) | 136 | 3.02 ± 0.48 | |  | 2 (4.2) | | 3 | 0.07 ± 0.05 |
| Females | 0.014 (20) | 12 (54.5) | 24 | 1.20 ± 0.29 | |  | 2 (9.1) | | 0 | 0 ± 0 |
| Males | 0.017 (25) | 20 (76.9) | 112 | 4.48 ± 0.73 | |  | 0 (0.0) | | 3 | 0.12 ± 0.09 |
| *Plot B* | 0.033 (33) | 28 (84.8) | 258 | 7.81 ± 1.43 | |  | 6 (18.2) | | 6 | 0.18 ± 0.07 |
| Females | 0.014 (14) | 10 (71.4) | 36 | 2.57 ± 1.03 | |  | 1 (7.1) | | 1 | 0.07 ± 0.07 |
| Males | 0.019 (19) | 18 (94.7) | 222 | 11.68 ± 1.95 | |  | 5 (26.3) | | 5 | 0.26 ± 0.10 |
| **Wood mice** | 0.021 (51) | **45 (86.5)** | **511** | **10.02 ± 1.86** | |  | **9 (17.3)** | | **22** | **0.43 ± 0.16** |
| *Plot A* | 0.021 (30) | 25 (80.6) | 177 | 5.90 ± 1.38 | |  | 5 (16.1) | | 12 | 0.40 ± 0.21 |
| Females | 0.010 (14) | 11 (78.6) | 58 | 4.14 ± 1.14 | |  | 2 (14.3) | | 2 | 0.14 ± 0.10 |
| Males | 0.011 (16) | 14 (82.4) | 119 | 7.44 ± 2.35 | |  | 3 (17.6) | | 10 | 0.63 ± 0.37 |
| *Plot B* | 0.021 (21) | 20 (95.2) | 334 | 15.90 ± 3.79 | |  | 4 (19.0) | | 10 | 0.48 ± 0.24 |
| Females | 0.006 (6) | 5 (83.3) | 56 | 9.33 ± 2.79 | |  | 0 (0.0) | | 0 | 0 ± 0 |
| Males | 0.015 (15) | 15 (100.0) | 278 | 18.53 ± 5.09 | |  | 4 (26.7) | | 10 | 0.67 ± 0.32 |
|  |  |  |  |  | |  |  | |  |  |
| **2014** | 0.507 (730) | **423 (56.4)** | **2252** | **3.08 ± 0.22** | |  | **24 (3.2)** | | **29** | **0.04 ± 0.01** |
| **Bank voles** | 0.308 (443) | **246 (54.5)** | **1275** | **2.88 ± 0.29** | |  | **9 (2.0)** | | **11** | **0.02 ± 0.01** |
| *Plot A* | 0.328 (236) | 110 (45.2) | 312 | 1.32 ± 0.15 | |  | 4 (1.6) | | 5 | 0.02 ± 0.01 |
| Females | 0.161 (116) | 44 (36.1) | 86 | 0.74 ± 0.11 | |  | 2 (1.6) | | 3 | 0.03 ± 0.02 |
| Males | 0.167 (120) | 66 (54.5) | 226 | 1.88 ± 0.26 | |  | 2 (1.7) | | 2 | 0.02 ± 0.01 |
| *Plot B* | 0.288 (207) | 136 (65.4) | 963 | 4.65 ± 0.59 | |  | 5 (2.4) | | 6 | 0.03 ± 0.01 |
| Females | 0.150 (108) | 61 (56.5) | 213 | 1.97 ± 0.41 | |  | 0 (0.0) | | 0 | 0 ± 0 |
| Males | 0.138 (99) | 75 (75.0) | 750 | 7.58 ± 1.07 | |  | 5 (5.0) | | 6 | 0.06 ± 0.03 |
| **Wood mice** | 0.199 (287) | **177 (59.2)** | **977** | **3.40 ± 0.32** | |  | **15 (5.0)** | | **18** | **0.06 ± 0.02** |
| *Plot A* | 0.197 (142) | 87 (56.5) | 528 | 3.72 ± 0.49 | |  | 10 (6.5) | | 13 | 0.09 ± 0.03 |
| Females | 0.100 (72) | 46 (56.8) | 178 | 2.47 ± 0.37 | |  | 5 (6.2) | | 6 | 0.08 ± 0.04 |
| Males | 0.097 (70) | 41 (56.2) | 350 | 5.00 ± 0.91 | |  | 5 (6.8) | | 7 | 0.10 ± 0.05 |
| *Plot B* | 0.201 (145) | 90 (62.1) | 449 | 3.10 ± 0.40 | |  | 5 (3.4) | | 5 | 0.03 ± 0.02 |
| Females | 0.085 (61) | 41 (67.2) | 213 | 3.49 ± 0.62 | |  | 4 (6.6) | | 4 | 0.07 ± 0.03 |
| Males | 0.117 (84) | 49 (58.3) | 236 | 2.81 ± 0.52 | |  | 1 (1.2) | | 1 | 0.01 ± 0.01 |

* No. of analysed rodents per trap-night, Plot A 2013 = 144 traps * 10 nights, plot B 2013 = 144 traps * 7 nights, plot A 2014 = 72 traps * 10 nights, plot B 2014 = 144 traps * 10 nights.

**Table S2.** Rodent infection rate, rodent infectivity, moulting success and contribution to DIN per year, rodent species, plot and rodent sex.

|  | Rodent infection rate  (infected/analysed rodents) | |  | Rodent infectivity  (infected/analysed larvae) | |  | Moulting success  (moulted/collected larvae) |  | Relative number of infected emerged nymphs * | |
| --- | --- | --- | --- | --- | --- | --- | --- | --- | --- | --- |
|  | *B. burgdorferi* | *B. miyamotoi* |  | *B. burgdorferi* | *B. miyamotoi* |  |  |  | *B. burgdorferi* | *B. miyamotoi* |
| **2013** | **0.35 (21/60)** | **0.05 (3/60)** |  | **0.46 (228/496)** | **0.32 (7/22)** |  | **0.96 (1086/1128)** |  | **0.058** | **0.00006** |
| **Bank voles** | **0.39 (15/38)** | **0.05 (2/38)** |  | **0.45 (110/245)** | **0.32 (7/22)** |  | **0.96 (491/512)** |  | **0.027** | **0.00003** |
| *Plot A* | 0.32 (7/22) | 0.05 (1/22) |  | 0.44 (17/39) | 0.2 (1/5) |  | 0.93 (113/121) |  | 0.012 | 0.00001 |
| Females | NA | NA |  | NA | NA |  | NA |  | NA | NA |
| Males | 0.32 (7/22) | 0.05 (1/22) |  | 0.44 (17/39) | 0.2 (1/5) |  | 0.93 (113/121) |  | 0.010 | 0.00001 |
| *Plot B* | 0.50 (8/16) | 0.06 (1/16) |  | 0.45 (93/206) | 0.35 (6/17) |  | 0.97 (378/391) |  | 0.056 | 0.00005 |
| Females | NA | NA |  | NA | NA |  | NA |  | NA | NA |
| Males | 0.50 (8/16) | 0.06 (1/16) |  | 0.45 (93/206) | 0.35 (6/17) |  | 0.97 (378/391) |  | 0.048 | 0.00005 |
| **Wood mice** | **0.27 (6/22)** | **0.05 (1/22)** |  | **0.47 (118/251)** | **(0/0)** |  | **0.97 (595/616)** |  | **0.026** | **0** |
| *Plot A* | 0.25 (3/12) | 0.08 (1/12) |  | 0.53 (46/87) | (0/0) |  | 0.97 (172/178) |  | 0.016 | 0 |
| Females | NA | NA |  | NA | NA |  | NA |  | NA | NA |
| Males | 0.25 (3/12) | 0.08 (1/12) |  | 0.53 (46/87) | (0/0) |  | 0.97 (172/178) |  | 0.010 | 0 |
| *Plot B* | 0.30 (3/10) | 0 (0/10) |  | 0.44 (72/164) | (0/0) |  | 0.97 (423/438) |  | 0.042 | 0 |
| Females | NA | NA |  | NA | NA |  | NA |  | NA | NA |
| Males | 0.30 (3/10) | 0 (0/10) |  | 0.44 (72/164) | (0/0) |  | 0.97 (423/438) |  | 0.035 | 0 |
|  |  |  |  |  |  |  |  |  |  |  |
| **2014** | **0.14 (100/705)** | **0.01 (8/705)** |  | **0.38 (94/246)** | **0.38 (3/8)** |  | **0.96 (1349/1410)** |  | **0.081** | **0.00006** |
| **Bank voles** | **0.15 (62/422)** | **0.01 (4/422)** |  | **0.20 (16/79)** | **(0/0)** |  | **0.93 (484/520)** |  | **0.025** | **0** |
| *Plot A* | 0.13 (32/238) | 0 (1/238) |  | 0.25 (2/8) | (0/0) |  | 0.91 (60/66) |  | 0.013 | 0 |
| Females | 0.11 (13/119) | 0 (0/119) |  | NA | (0/0) |  | NA |  | NA | NA |
| Males | 0.16 (19/119) | 0.01 (1/119) |  | 0.25 (2/8) | (0/0) |  | 0.91 (60/66) |  | 0.011 | 0 |
| *Plot B* | 0.16 (30/184) | 0.02 (3/184) |  | 0.20 (14/71) | (0/0) |  | 0.93 (424/454) |  | 0.040 | 0 |
| Females | 0.14 (13/90) | 0.02 (2/90) |  | NA | (0/0) |  | NA |  | NA | NA |
| Males | 0.18 (17/94) | 0.01 (1/94) |  | 0.20 (14/71) | (0/0) |  | 0.93 (424/454) |  | 0.035 | 0 |
| **Wood mice** | **0.13 (38/283)** | **0.01 (4/283)** |  | **0.47 (78/167)** | **0.38 (3/8)** |  | **0.97 (865/890)** |  | **0.041** | **0.00004** |
| *Plot A* | 0.13 (20/149) | 0.01 (1/149) |  | 0.61 (51/84) | 0.38 (3/8) |  | 0.96 (436/452) |  | 0.058 | 0.00002 |
| Females | 0.09 (7/78) | 0 (0/78) |  | NA | (0/0) |  | NA |  | NA | NA |
| Males | 0.18 (13/71) | 0.01 (1/71) |  | 0.61 (51/84) | 0.38 (3/8) |  | 0.96 (436/452) |  | 0.052 | 0.00003 |
| *Plot B* | 0.13 (18/134) | 0.02 (3/134) |  | 0.33 (27/83) | (0/0) |  | 0.98 (429/438) |  | 0.027 | 0 |
| Females | 0.04 (2/51) | 0.04 (2/51) |  | NA | (0/0) |  | NA |  | NA | NA |
| Males | 0.19 (16/83) | 0.01 (1/83) |  | 0.33 (27/83) | (0/0) |  | 0.98 (429/438) |  | 0.020 | 0 |

NA = not analysed

* calculated as: relative rodent density (Table 1) * mean larval tick burden (Table 1) * rodent infection
